# Genome-wide Identification, Expression, and Functional Analysis of *MdMSI* Genes in Apples (*Malus domestica* Borkh.)

**DOI:** 10.3389/fgene.2022.846321

**Published:** 2022-03-03

**Authors:** Daru Wang, Xun Wang, Chunling Zhang, Kuo Yang, Xinjie Wang, Jianying Cui, Dandan Liu, Chunxiang You

**Affiliations:** ^1^ National Key Laboratory of Crop Biology, National Research Center for Apple Engineering and Technology, College of Horticulture Science and Engineering, Shandong Agricultural University, Taian, China; ^2^ College of Agriculture, Yunnan University, Kunming, China

**Keywords:** apple, MSI, expression pattern, salt stress, phylogenetic tree

## Abstract

The multicopy suppressor of IRA (MSI) is a subfamily of WD40 repeat proteins, which is widely involved in plant growth and development. In order to explore the function of *MdMSI* members in abiotic stress, we identified eight *MSI* gene family members from the *Malus* × *domestica* reference genome. They were distributed on six chromosomes, and they had similar secondary and tertiary structures. We found a variety of regulatory elements in response to hormones and abiotic stress in *MdMSI* promoters. Through qRT-PCR analysis, it was revealed that *MdMSIs* were expressed in all tissues, especially in roots. The analysis results also revealed that the expression of *MdMSIs* was induced in varying degrees under salt, drought stress, and ABA treatments. Furthermore, we obtained the overexpression of *MdMSI1-1* transgenic apple calli and *Arabidopsis*. The overexpression of *MdMSI1-1* in calli and *Arabidopsis* played a negative regulatory role in salt stress response. Our work laid a foundation for further verifying the function of *MSI* genes under abiotic stress in apples.

## Introduction

WD40 repeat protein is generally composed of 4–10 corresponding WD40 domains, of which the WD40 domain is composed of about 40 conserved amino acids, and its N-terminal is the glycine–histidine dimer peptide. WD40 protein is the most studied in eukaryotes and plays a variety of important functions, such as embryogenesis and gamete formation, flower development, and flowering process (Jiang et al., 2009; Jiang et al., 2011; Pazhouhandeh et al., 2011). Some members can respond to abiotic stress and hormone induction (Cheng et al., 2014; Qi et al., 2014; Liu et al., 2018).

The multicopy suppressor of IRA (MSI) is a kind of WD40 repeat protein, which has similar and specific functions in different plants. *MSI1* is screened for the first time in *Saccharomyces cerevisiae* and is determined to negatively regulate the RAS-mediated cAMP pathway (Ruggieri et al., 1989). In previous studies, the *MSI* family is widely reported in plant reproductive development, such as male and female gamete development, endosperm development, regulating flowering, and low-temperature vernalization (Kaya et al., 2001; Rossi et al., 2001; Guitton and Berger 2005; Pazhouhandeh et al., 2011; Derkacheva et al., 2013). In addition, *MSI* genes play an important role in participating in some abiotic stresses during plant growth and development (Jeon and Kim 2011; Kenzior and Folk 2015; Cui et al., 2022). There are five members of this family in *Arabidopsis* (Hennig et al., 2005), among which *AtMSI1* plays a negative regulatory role in salt and drought stress (Alexandre et al., 2009; Mehdi et al., 2016). *AtMSI4* plays an important role in controlling of flowering and salt stress (Kenzior and Folk 2015). Other studies have shown that *AtMSI4/FVE* plays a role in cold resistance (Kim et al., 2004). At present, there are few studies on *AtMSI*2 and *AtMSI*3, and it is uncertain about what kind of function they play in *Arabidopsis* (Shen et al., 2019). Moreover, GmFVE can interact with GmNFYA and play a negative regulatory role in salt stress (Lu et al., 2021). Some members of the *MSIL* family in longan have a variety of abiotic stress response elements. It is considered that some members of the *MSIL* gene family may participate in a variety of abiotic stresses (Shen et al., 2019).

The apple is one of the fruits with the largest planting area in the world (An et al., 2016; Wang et al., 2019). With the increasing deterioration of the global environment, plants will encounter more and more complex stresses in the growth process. Salt and drought stress have great effects on plant growth and development (Manickavelu et al., 2006; Jaleel et al., 2009). Salt stress will cause ion and osmotic stress to plants, and drought stress will reduce the plant photosynthetic rate and photosynthetic electron transfer rate (Tian et al., 2015; Sequera-Mutiozabal et al., 2016).

In apples, the *MSI* gene family has not been systematically investigated. In this study, the whole genome identification, systematic prediction and analysis of gene and protein levels, tissue expression, and abiotic stress changes of the *MdMSI* gene family were carried out. In addition, we further verified the salt intolerant function of *MdMSI-1* by genetic transformation of apple calli and *Arabidopsis*. It provides a theoretical basis for studying the mechanism of the *MdMSI* gene family in the process of abiotic stress in apples.

## Materials and Methods

### Plant Materials and Growth Conditions

The test plant materials were obtained from 12-year-old “Royal Gala” apple (*Malus domestica* Borkh. “Royal Gala”) trees cultivated at the Shandong Agricultural University experimental station and from 1-month-old self-rooted apple seedlings [*Malus hupehensis* (Pamp.) Rehd. “pinyiensis.”] cultivated in the laboratory of molecular biology of fruit trees of the Academy of Horticultural Science and Engineering, Shandong Agricultural University. After taking the roots, stems, leaves, flowers (initial flowering), peels, and pulps of “Royal Gala,” the samples were quickly added in liquid nitrogen and timely transferred to an ultra-low temperature refrigerator for storage for subsequent test operations.

The apple shoot cultures, derived from “Royal Gala,” were stored at 24°C on MS solid medium (containing 0.8% agar, 0.2 mg/L NAA, 0.5 mg/L 6-BA, and 0.1 mg/L GA3) under a photoperiod (8/16 h dark/light) for 25 days. To obtain self-rooted plantlets, the 4-week-old shoot cultures were transferred to a root-inducing MS solid medium containing 0.2 mg/L IAA. For gene expression analysis, 25-day-old self-rooted apple seedlings were treated with 150 mM NaCl, 10% PEG 6000, and 100 μM ABA. Apple seedlings were sampled at different time points after treating for 0, 1, 3, 6, and 12 h and were quickly placed in liquid nitrogen and stored in an ultra-low temperature refrigerator for subsequent experiments (Yamaguchi-Shinozaki and Shinozaki 1994).

“Orin” apple calli were grown on MS solid medium supplemented with 0.8% agar, 0.4 mg/L 6-BA, and 1.5 mg/L 2, 4-dichlorophenoxyacetic acid (2,4-D) at 25°C in the dark. After 15 days of growth, calli (WT *and MdMSI1-1-OX*) were transferred to the MS solid medium supplemented with 0 or 100 mM NaCl and placed in the dark for 16 days. *Arabidopsis thaliana* (Columbia) seeds were germinated on the MS solid medium at 22°C in a photoperiod (16 h/8 h, light/dark). The 3-day-old *Arabidopsis* seedlings (WT *and MdMSI1-1-OE*) were transferred to the MS solid medium that supplied with 0 or 100 mM NaCl for 14 days. After treatment, we measured the MDA content of calli following the study by (Ma et al., 2017) and the relative electronic conductivity level of *Arabidopsis* following the study by (Hu et al., 2013).

### Construction of the MdMSI1-1 Expression Plasmid

The full-length cDNA of *MdMSI1-1* was cloned into the PRI-101 (35S promoter, GFP) vector to obtain its overexpression plasmid. The primer pairs *MdMSI1-1-F* (5′-ATGGGCAAAGAC -3′)/−R and (5′- AGGCTTTGCCGGTTC -3′) were used to amplify the full-length *MdMSI1-1*.

### Genetic Transformation

Apple calli were infected by *Agrobacterium*-mediated transformation to obtain *MdMSI1-1* overexpression transgenic apple calli (*MdMSI1-1-OX*) (An et al., 2016). The transgenic *Arabidopsis* (*MdMSI1-1-OE*) were obtained by using the floral dip transformation method (Yang et al., 2021).

### Identification and Basic Properties of MSI Family Members in the Apple

The apple “GDDH13” reference genome is from the Apple Genome and Epigenome database (GDDH13_1-1, https://iris.angers.inra.fr/gddh13/) (Daccord et al., 2017). *Arabidopsis* MSI proteins are from the NCBI (https://www.ncbi.nlm.nih.gov/). The apple MSIs were searched by using AtMSI sequences (BLASTp). In addition, apple MSIs were obtained by the HMM method with the E-value (10e^−5^) (http://pfam.xfam.org/) (Finn et al., 2014). The domains (CAF1C_H4-bd, WD40) were analyzed and confirmed by online software SMART (http://smart.embl-heidelberg.de/). The members of apple MSIs were finally obtained. The number of amino acids, molecular weight, isoelectric point, and other physical and chemical properties of *MdMSI* gene family members were analyzed by using ProtParam in online software EXPASY (https://web.expasy.org/protparam/). The subcellular localization of *MdMSIs* was predicted by online software WoLF PSORT (https://www.genscript.com/wolf-psort.html) (Horton et al., 2007).

### Domain Analysis and Multi-Sequence Alignment of MdMSIs

MdMSI protein sequence alignment was performed by online software Clustal Omega (https://www.ebi.ac.uk/Tools/msa/clustalo/) and visualized by Jalview (Jalview 2.11.1.4).

### Phylogenetic Tree and Conserved Motif Analysis of MdMSIs

MdMSI protein sequences were downloaded from the GDDH13 website, AtMSIs from the TAIR (http://www.Arabidopsis.org/), and OsMSIs from the Phytozome (https://phytozome.jgi.doe.gov/pz/portal.html). All MSI protein sequences of the apple, *Arabidopsis*, and rice were constructed using a phylogenetic tree by software Mega7 (Kumar et al., 2016). The phylogenetic tree was constructed by using the NJ method (Bootstrap set to 1,000), and 10 conserved motifs of MdMSIs and AtMSIs were analyzed by online software MEME (http://meme-suite.org/) (Bailey et al., 2006).

### MdMSI Protein Structure Prediction and Protein Interaction Analysis

The secondary structures of MdMSI proteins were predicted by online software SOPMA (https://npsa-prabi.ibcp.fr/cgi-bin/npsa_automat.pl?page=npsa_sopma.html). The tertiary structures of MdMSI proteins were predicted by homology modeling of online software Phyre2 (http://www.sbg.bio.ic.ac.uk/phyre2/html/page.cgi? id=index) (Kelley et al., 2015).

The interaction of AtMSI member-related proteins was predicted by online software STRING (https://stringdb.org) (Szklarczyk et al., 2019), and then, the related homologous apple *MSI* members were matched.

### Chromosome Mapping and Gene Structure Analysis of MSIs in Apples

The chromosome position of apple *MSI* family members was visually analyzed by online software MG2C (http://mg2c.iask.in/mg2c_v2.1/). Using gene annotation information, the gene structure of *MSI* family members was analyzed by online software GSDS 2.0 (http://gsds.gao-lab.org/) (Hu et al., 2015).

### Promoter Analysis of MdMSIs

The promoter region of about 2000 bp upstream of the apple *MSI* gene was submitted to the online software PlantCARE (http://bioinformatics.psb.ugent.be/webtools/plantcare/html/) (Lescot et al., 2002) for *cis*-acting element analysis.

### Quantitative Real-Time PCR Analysis

RNA was extracted from the apple material using the RNA plant plus Kit (Tiangen, Beijing), cDNA was obtained by reverse transcription of the extracted RNA using the primescript RT reagent kit a with gDNA eraser (Takara, Dalian), qRT-PCR was performed on an iCycler iQ5 system (Bio-RAD) instrument, and data on relative gene expressions were analyzed by 2^−ΔΔCT^ methods. The quantitative primer design for the *MSI* genes in the apple was performed by using online software Primer3Plus (http://primer3plus.com/cgi-bin/dev/primer3plus.cgi) (Untergasser et al., 2007), as shown in Supplementary Table S1.

### Statistical Analysis

We performed data significance analysis by DPS software. According to the Tukey-Kramer test, it indicated that different lowercase letters represent significant differences. Each experiment was repeated at least three times.

## Results

### Identification and Basic Information of MdMSIs

After stringent dereplication screening using the online software SMART and Pfam with *AtMSIs* as the query sequence, eight *MdMSI* members were finally identified from the “Golden Delicious” apple genome sequence, which were sequentially named *MdMSI1-1* (MD02G1228600), *MdMSI1-2* (MD07G1083900), *MdMSI2* (MD15G1206400), *MdMSI3-1* (MD05G1121700), *MdMSI3-2* (MD10G1124300), *MdMSI4-1* (MD02G1079600), *MdMSI4-2* (MD09G1193500), and *MdMSI4-3* (MD15G1207100) based on their homology to *Arabidopsis MSI* family genes. Using the genome annotation file and protein analysis website, the gene length, coding sequence, amino acid number, molecular weight, isoelectric point (*pI*), and subcellular localization of *MdMSI* genes were statistically analyzed. It was found that the length of *MdMSI* genes ranged from 2,673 to 5,972 bp: the shortest was *MdMSI3-2* and the longest was *MdMSI4-2*. The length of the coding sequence ranged from 1,080 to 1,557 bp: the shortest was *MdMSI3-2* and the longest was *MdMSI4-1*. The number range of amino acids was 359–518, the molecular weight was 40,171.82–57,353.08 Da, the isoelectric point was 4.60–6.08, and the *pI* was less than 7. By using online software WoLF PSORT, the subcellular localization of *MdMSIs* was predicted to be all in the nucleus (Table 1).

**TABLE 1:** Identification of MdMSIs.

Gene	Accession number	Gene length	CDS length	Size of aa	MW(Da)	*pI*	Subcellular localization
*MdMSI1-1*	MD02G1228600	4,498	1,269	422	48,206.97	4.72	Nucleus
*MdMSI1-2*	MD07G1083900	4,750	1,269	422	48,129.85	4.71	Nucleus
*MdMSI2*	MD15G1206400	3,151	1,422	473	51,837.64	5.01	Nucleus
*MdMSI3-1*	MD05G1121700	3,152	1,224	407	45,925.03	4.60	Nucleus
*MdMSI3-2*	MD10G1124300	2,673	1,080	359	40,171.82	4.76	Nucleus
*MdMSI4-1*	MD02G1079600	5,592	1,557	518	57,353.08	5.78	Nucleus
*MdMSI4-2*	MD09G1193500	5,972	1,368	455	49,961.70	5.75	Nucleus
*MdMSI4-3*	MD15G1207100	5,472	1,497	498	55,145.80	6.08	Nucleus

### Protein Domain Analysis and Multi-Sequence Alignment of MdMSIs

**FIGURE 1:**
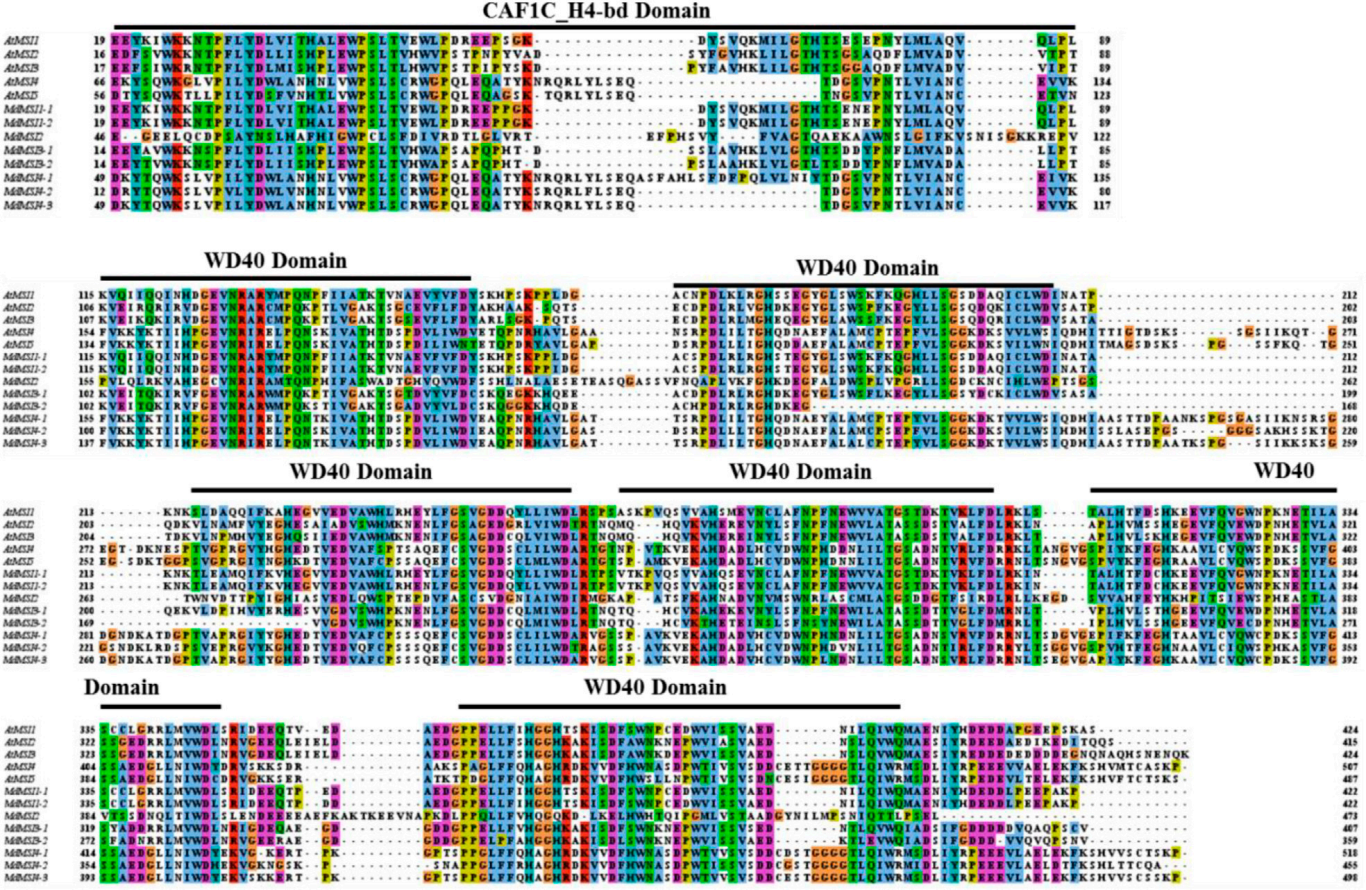
Core conserved domain of MdMSI proteins.

In the process of protein domain query by online software SMART, it was found that the eight MSI proteins of apple and the five MSI proteins of *Arabidopsis* contain two typical domains: one was the CAF1C_H4-bd domain, and the other was a typical WD40 domain (Figure 1). The MSI protein sequences of *Arabidopsis* and apple were compared by online software Clustal Omega and visualized by using the software tool Jalview 2.11.1.4. *Arabidopsis* and apple MSI proteins had only one highly conserved CAF1C_H4-bd domain. Another WD40 domain was composed of about 40 amino acids.

### Evolutionary Tree and Motif Analysis of MdMSIs

**FIGURE 2:**
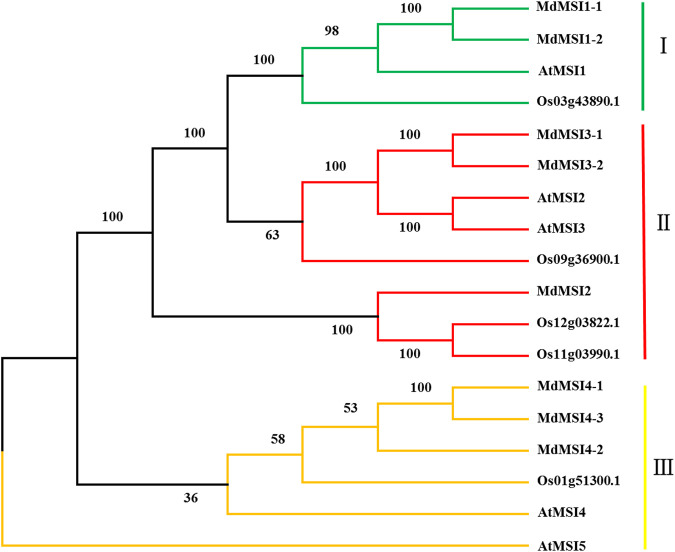
Phylogenetic tree of MdMSI proteins.

The phylogenetic tree was constructed by using apple, *Arabidopsis*, and rice MSIs, and the apple MSI was grouped according to the classification of *Arabidopsis* MSI. MSI were divided into three categories, MdMSI1-1 and MdMSI1-2 were divided into one category (I); MdMSI2, MdMSI3-1, and MdMSI3-2 were divided into one category (II); and MdMSI4-1, MdMSI4-2, and MdMSI4-3 were divided into one category (III) (Figure 2).

**FIGURE 3:**
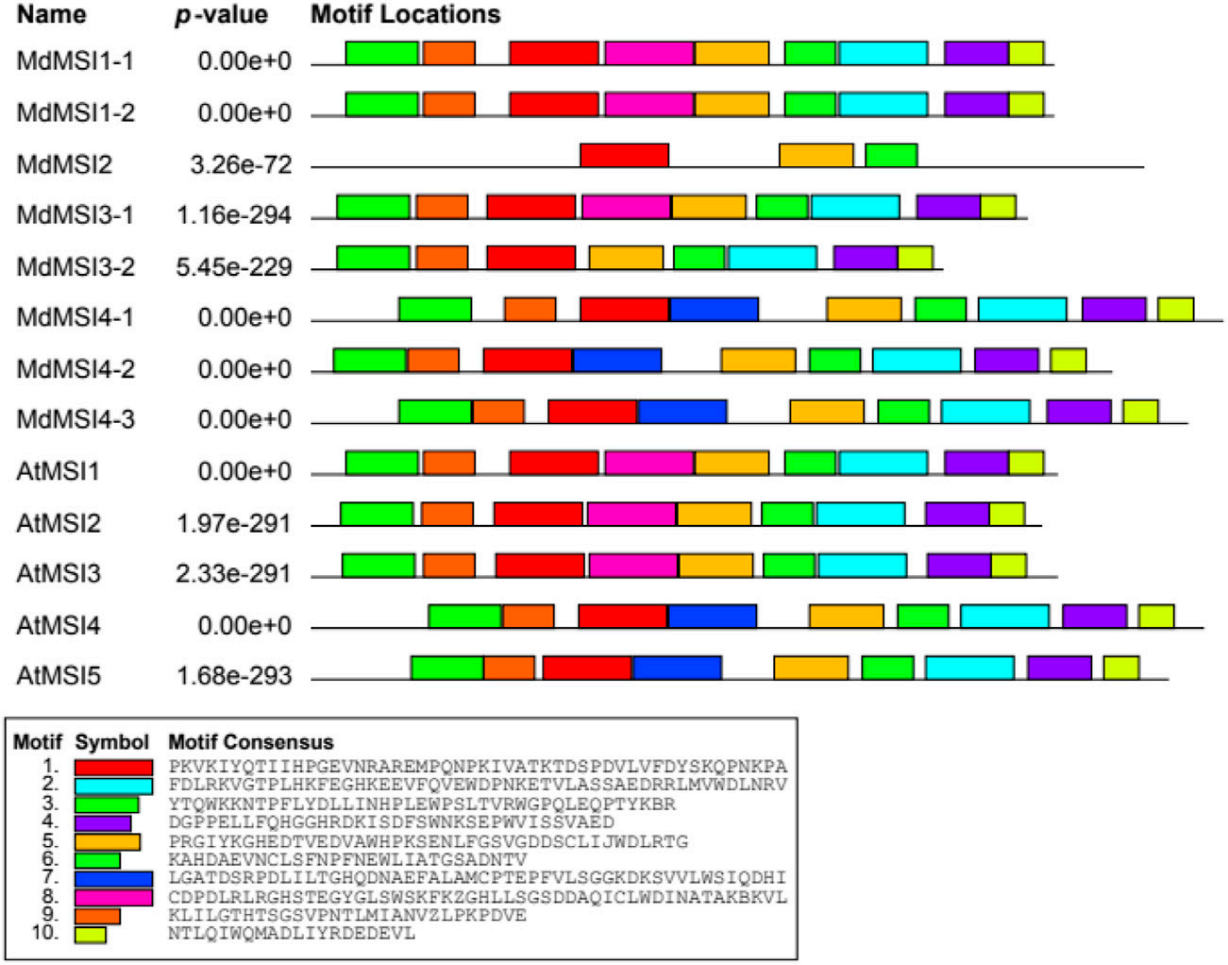
Conserved motifs of MdMSI proteins in apple and *Arabidopsis.*

Using the online software MEME, we predicted 10 motifs in the MdMSI and AtMSI proteins (Figure 3). The motif distribution of MSI proteins was similar. All MSI proteins contain motifs 1, 5, and 6. However, there are also differences in the composition of MSI protein motifs in different groups of the evolutionary tree (Figure 2). For example, motif 7 was detected only in members of (III) (MdMSI4-1, MdMSI4-2, and MdMSI4-3 in apple and AtMSI4 and AtMSI5 in *Arabidopsis*).

### Protein Structure Prediction of MdMSIs

**FIGURE 4:**
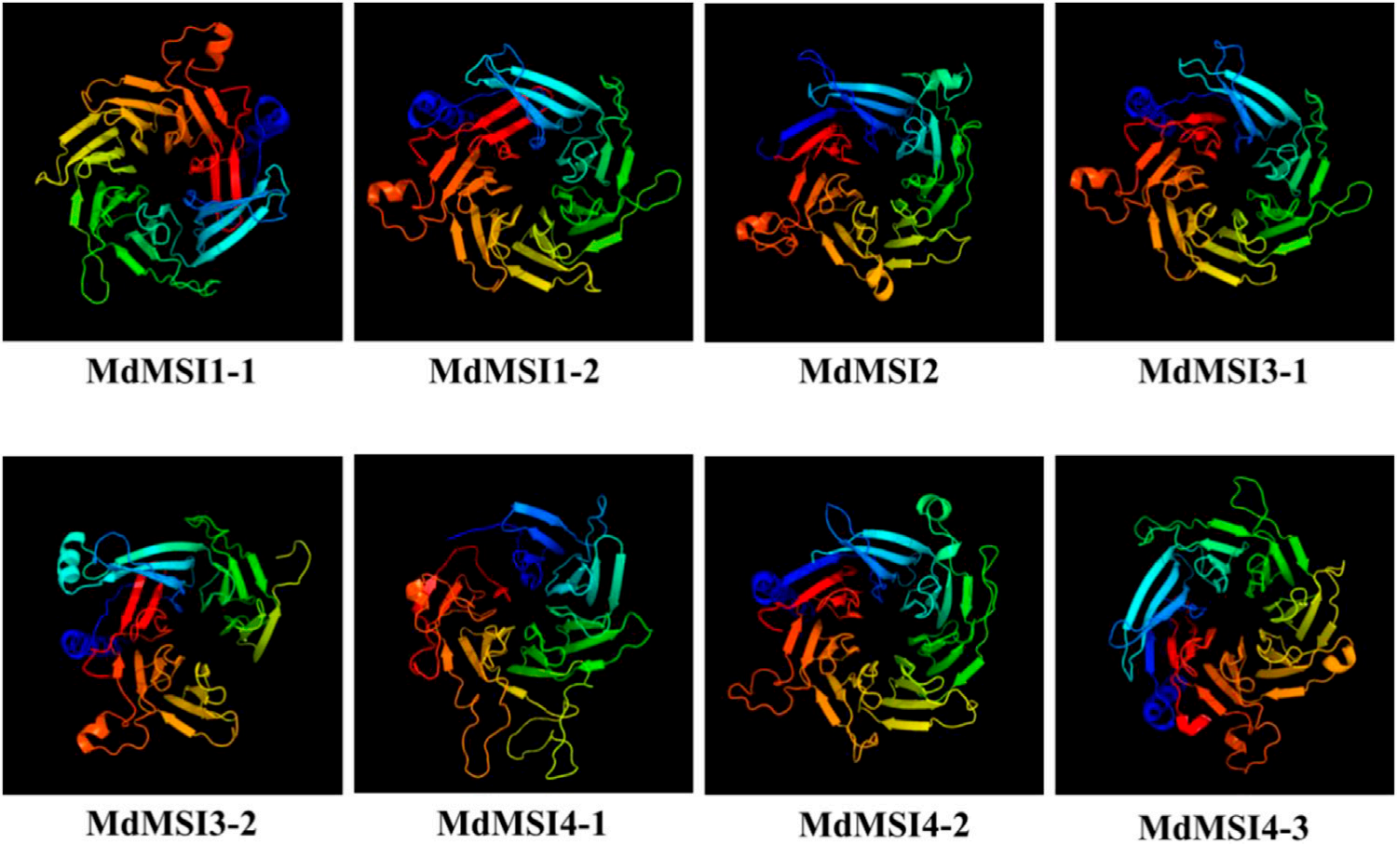
Three-dimensional structure of MdMSI proteins.

The secondary structure of MdMSI protein was compared. The largest proportion of the secondary structure of eight proteins was random coil, followed by α-helix, and the smallest proportion was β-turn. Moreover, these proteins had roughly similar proportions of the four secondary structure elements (Table 2). The online software Phyre2 was used for the homology model to predict MdMSI families, and the results showed that the tertiary structure of the protein-conserved region of the MdMSI family was very consistent (Figure 4). The special structure was often related to its function, indicating that their function was very likely to be similar.

**TABLE 2:** Secondary structure of MdMSIs.

Protein	α-helix (%)	β-turn (%)	Random coil (%)	Extend strand (%)
MdMSI1-1	15.17	5.69	50.00	29.15
MdMSI1-2	14.22	4.98	52.13	28.67
MdMSI2	13.53	6.55	53.91	26.00
MdMSI3-1	13.02	7.13	48.89	30.96
MdMSI3-2	15.60	5.85	48.19	30.36
MdMSI4-1	16.99	3.67	54.05	25.29
MdMSI4-2	13.19	3.08	54.51	29.23
MdMSI4-3	17.27	3.61	52.61	26.51

### Construction of MdMSI Protein Interaction Protein Network

**FIGURE 5:**
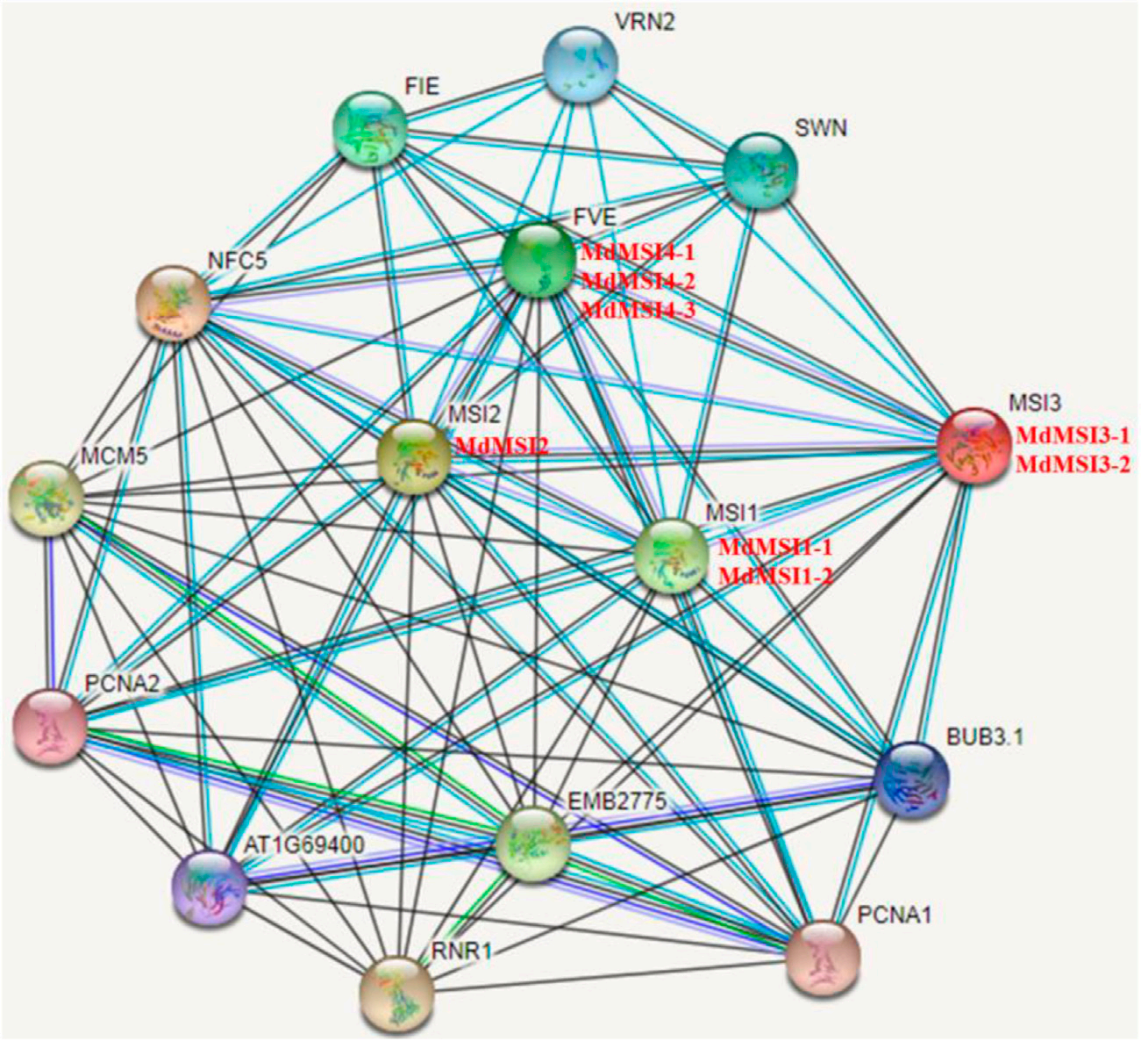
Prediction of the interacted protein network of MdMSIs.

By using online software STRING, the protein network and potential function of the MdMSI protein interaction were predicted. MdMSI1-1 and MdMSI1-2 correspond to AtMSI1; MdMSI2 corresponds to AtMSI2; MdMSI3-1 and MdMSI3-2 correspond to AtMSI3; and MdMSI4-1, MdMSI4-2, and MdMSI4-3 correspond to AtFVE (Figure 5). According to the predicted results of the *Arabidopsis* MSI protein interaction network, AtMSI was associated with FIE protein, SWN protein, VRN2 protein, RNR1 protein, PCNA1 protein, and PCNA2 protein. In addition to interacting with reproductive development-related proteins, such as the FIE protein, SWN protein, and VRN2 protein, it was also associated with stress-related proteins, such as chloroplast posttranscriptional regulation-related protein RNR1 and PCNA1/2 protein interaction, which played an important role in DNA damage response. It showed that the MSI protein was regulated by multiple protein interactions.

### Chromosome Location and Gene Structure Analysis of MSIs in Apple

**FIGURE 6:**
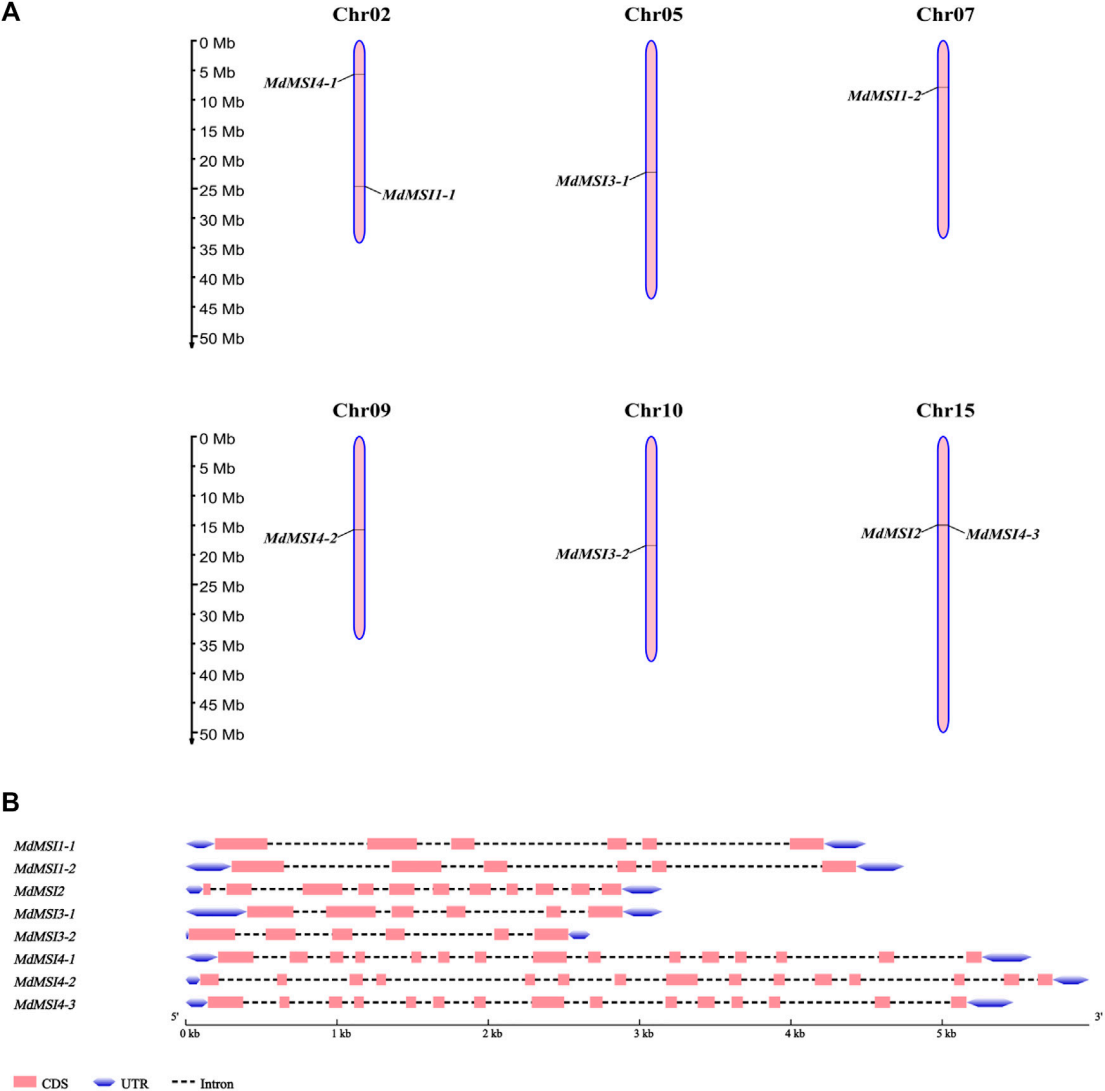
Genomic location **(A)** and structure **(B)** of *MdMSIs.*

Chromosome location and gene structure analysis of *MdMSI* genes showed that they were unevenly distributed on six chromosomes, of which only one gene was distributed on chr05, chr07, chr09, and chr10, which were *MdMSI3-1*, *MdMSI1-2*, *MdMSI4-2*, and *MdMSI3-2* respectively. There were two genes on chr02 and chr15, respectively, *MdMSI1-1* and *MdMSI4-1*, *MdMSI2* and *MdMSI4-3* (Figure 6A). The online software GSDS 2.0 was used to visually analyze the gene structure. The number of exons of the gene family was 6–15, and the number of introns was 5–14. It was very interesting that *MdMSI1-1* and *MdMSI1-2*, *MdMSI3-1*, and *MdMSI3-2* corresponding to *AtMSI1* and *AtMSI3*, respectively, had six exons and five introns (Figure 6B). According to the analysis of the aforementioned apple *MSI* phylogenetic tree, it was speculated that the two groups of genes were likely to have similar functions. In addition, the three genes corresponding to *AtMSI4* also had the same exon and intron, and their numbers are 15 and 14, respectively.

### *Cis*-Element Analysis of MdMSI

The promoter of *MSI* genes in apples was analyzed (Table 3). It was detected that there were a large number of *cis*-acting elements in response to hormones and stress, such as the ABRE element in response to abscisic acid, CGTCA of jasmonic acid, ERE of ethylene, P-box and GARE motif of gibberellin, TCA of salicylic acid, and TGA of auxin, and were in response to hypoxia, MBS in drought, TC-rich repeats of defense and stress, etc. In conclusion, it was speculated that apple *MSI* may play an important role in abiotic stress.

**TABLE 3:** *Cis*-acting elements of *MdMSI* promoters.

Gene	ABRE	ACE	ARE	Box4	CGTCA	ERE	LTR	MBS	P-box	GARE-motif	TC-rich repeats	TCA	TGA	W-box
MdMSI1-1		1/0	1/2	0/1	0/1	2/0	1/1	1/1	0/2		1/0	0/1	1/0	1/1
MdMSI1-2	3/0		1/4	0/1	2/0	0/1	1/0	0/2			1/0	0/1		
MdMSI2	2/0		2/0	1/0			1/0	0/1				1/0	3/0	
MdMSI3-1	9/3	1/0	1/0	0/3	1/1	1/1	2/0	1/1	0/1		0/1			0/1
MdMSI3-2	2/2		0/1	1/0	1/3		1/0				1/0	0/1		2/0
MdMSI4-1	1/0		1/3	0/1	1/1		2/0	2/0				1/1	0/1	0/1
MdMSI4-2			4/0	0/1	1/0	1/0				0/1				
MdMSI4-3	0/2		1/2	0/5	1/0	2/0	1/0	1/0	2/0					

### Expression of *MdMSI* Genes in Different Tissues

**FIGURE 7:**
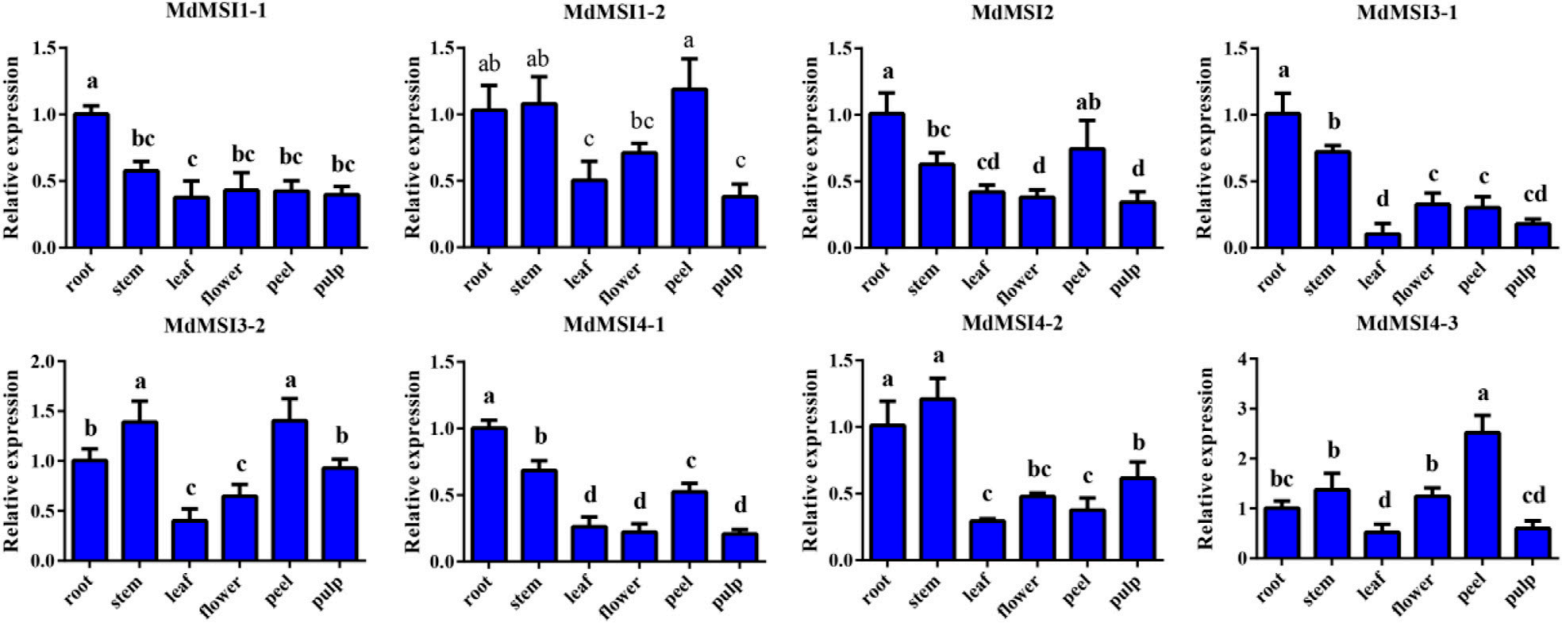
Relative expression levels of *MdMSIs* in different tissues. Different lowercase letters represent a significant difference (*p* < 0.05).

In order to explore the temporal and spatial expression of *MdMSIs* in apple, they were analyzed by qRT PCR in roots, stems, leaves, flowers, peels, and pulps. *MdMSIs* were expressed in all tested tissues (Figure 7). *MdMSI1-1*, *MdMSI2*, *MdMSI3-1*, and *MdMSI4-1* were the most expressed in roots. The expression of *MdMSI1-1* in roots was about two times higher than that in other tissues. Moreover, *MdMSI1-2*, *MdMSI3-2*, and *MdMSI4-3* were highly expressed in stems and peels. They all had a low expression level in the leaf.

### Expression Analysis of MdMSIs Under Abiotic Stress and Abscisic Acid

**FIGURE 8:**
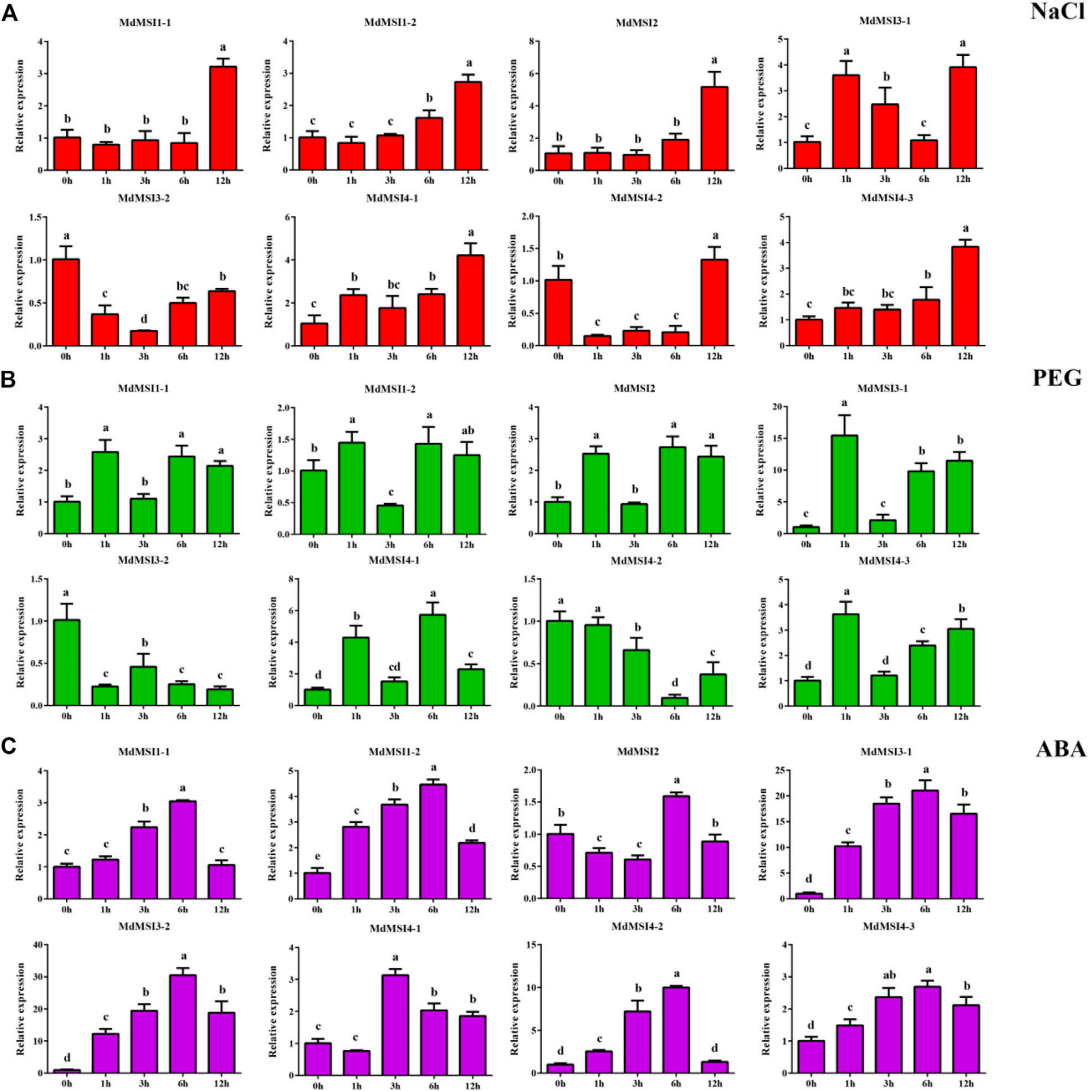
Expression analysis of *MdMSI* genes under different stress conditions. Expression of *MdMSIs* under **(A)** NaCl, **(B)** PEG, and **(C)** ABA conditions. Different lowercase letters represent a significant difference (*p* < 0.05).

In order to study the response of apple *MSI* genes to abiotic stress, the seedlings of apple were treated with salt and drought. After salt stress, the relative expression levels of the other seven genes reached the maximum at 12 h, except *MdMSI3-2* (Figure 8A)*.* In addition, it was very interesting that the relative expression of the *MdMSI* gene family decreased first and then increased after salt stress treatment, except *MdMSI3-1* and *MdMSI4-1*. After drought treatment, except *MdMSI3-2* and *MdMSI4-2*, the overall expression of the other six genes in the *MdMSI* gene family showed an upward trend (Figure 8B). In addition, many *cis*-elements related to hormones were found in the analysis of the apple *MSI* promoter. Among them, the number and range of ABA were the most extensive in the *MdMSI* gene family. Therefore, the seedlings of apple were also treated with ABA. The expressions of *MdMSI1-1*, *MdMSI1-2*, *MdMSI3-1*, *MdMSI3-2*, *MdMSI4-2*, and *MdMSI4-3* were similar, and they first showed an upward trend, peaked at 6 h, and then decreased (Figure 8C). The expression of *MdMSI4-1* reached the highest levels at 3 h.

### MdMSI1-1 Overexpression Increases Sensitivity to Salt Stress in Transgenic Apple Calli and *Arabidopsis*

**FIGURE 9:**
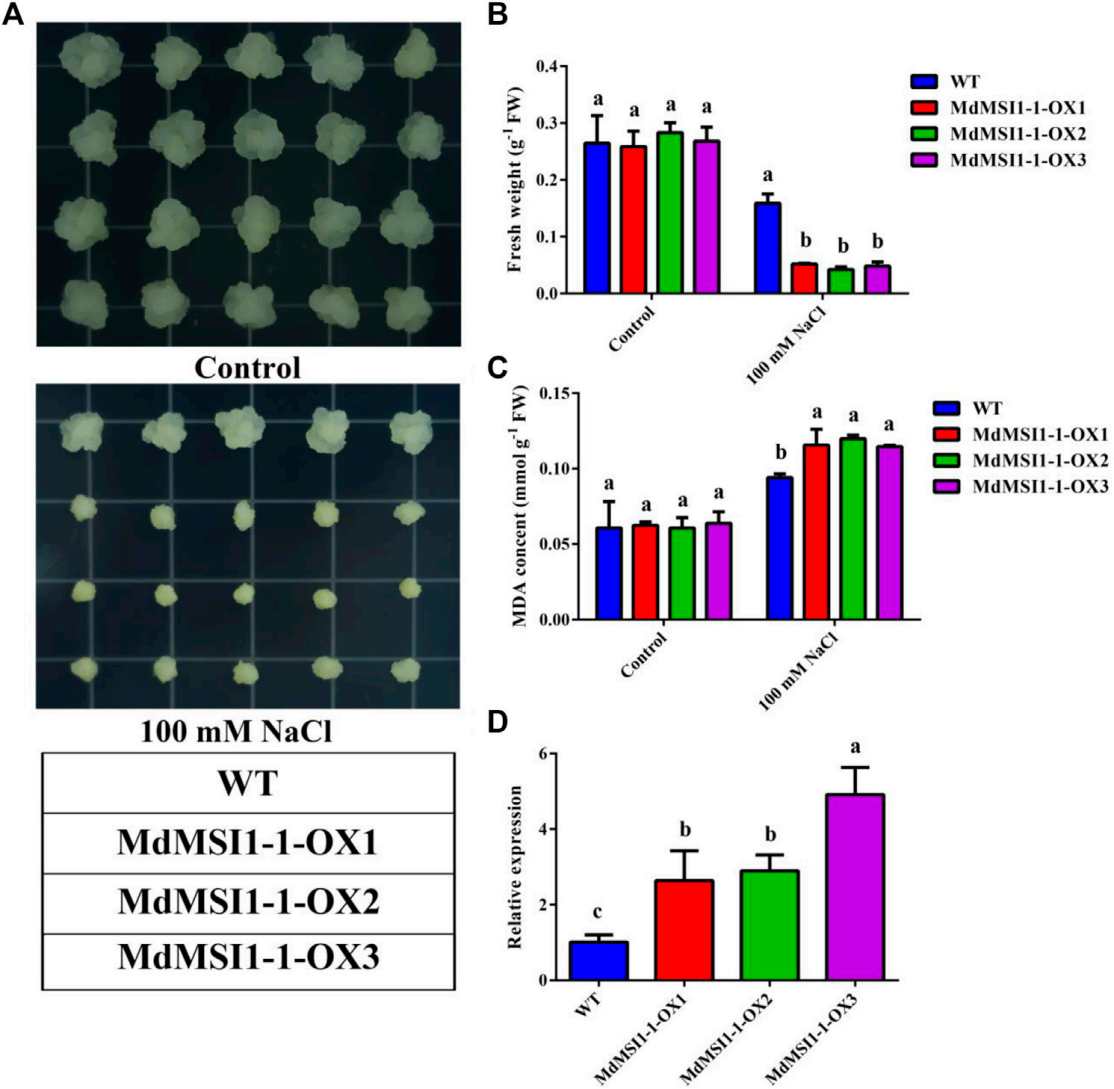
*MdMSI1-1* increased sensitivity to salt stress in apple calli. **(A)** The phenotypes of 16-day-old WT and *MdMSI1-1-OX* calli in MS, or MS + 100 mM NaCl, respectively. **(B)** fresh weight and **(C)** MDA content in apple calli after treatment (MS, or MS + 100 mM NaCl). **(D)** The expression level of *MdMSI1-1* in WT and *MdMSI1-1-OX* calli. Different lowercase letters represent a significant difference (*p* < 0.05).

Among the *MdMSIs* promoters, a large number of *cis*-elements related to hormones and abiotic stress were identified in *MdMSIs* promoters, indicating that the family genes may be involved in abiotic stress response (Table 3). There were a lot of abiotic stress-related elements in the *MdMSI1-1* promoter sequence, and its expression level was significantly affected by salt stress. Therefore, we predicted that it was very likely to participate in salt stress. We obtained three transgenic apple callus lines of overexpressed *MdMSI1-1* (*MdMSI1-1-OX1*, *MdMSI1-1-OX2*, and *MdMSI1-1-OX3*) (Figure 9D). Under the control condition, there was no significant difference in fresh weight and MDA between WT and *MdMSI1-1-OX*, but under the salt treatment condition, the fresh weight of *MdMSI1-1-OX* was significantly lower than WT, and MDA was significantly higher than WT (Figures 9A–C). The results showed that *MdMSI1-1* negatively regulated salt stress in apple.

**FIGURE 10:**
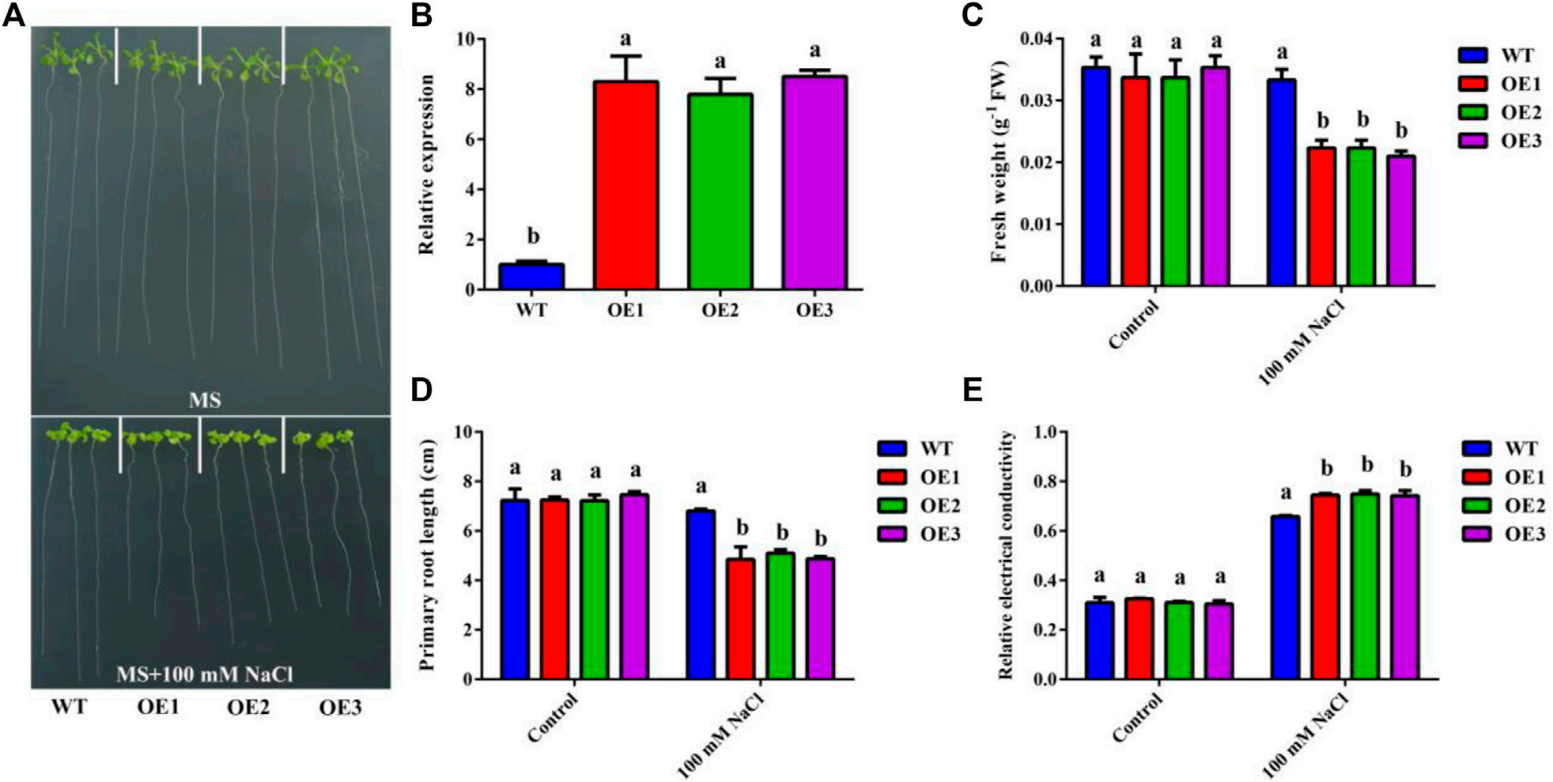
Ectopic expression of *MdMSI1-1* negatively regulated salt tolerance in *Arabidopsis*. **(A)** The phenotypes of *Arabidopsis* seedlings (WT, and *MdMSI1-1-OE*) treated with MS, or MS + 100 mM NaCl treatment. **(B)** Expression of *MdMSI1-1*in *Arabidopsis* (WT, and *MdMSI1-1-OE*). **(C)** fresh weight, **(D)** primary root length, and **(E)** relative electronic conductivity in WT and transgenic *Arabidopsis* after treatment (MS, or MS + 100 mM NaCl). Different lowercase letters represent a significant difference (*p* < 0.05).

In order to further verify the function of *MdMSI1-1* in salt stress, we obtained three *MdMSI1-1* overexpressions in *Arabidopsis* (OE1, OE2, and OE3) (Figure 10B). We treated 3-day-old *Arabidopsis* seedlings (WT and *MdMSI1-1-OE*) with 100 mM NaCl and found that *MdMSI1-1-OE* had lighter fresh weight and shorter primary root length than WT (Figures 10A,C,D). Moreover, we detected the relative electronic conductivity in *Arabidopsis* (Figure 10E). The relative electronic conductivity of *MdMSI1-1-OE* was higher than that of WT. These results showed that *MdMSI1-1* increased the sensitivity of apple calli and *Arabidopsis* under salt stress.

## Discussion

WD40 repeat proteins widely exist in eukaryotes (Janda et al., 1996), and its function has been reported in many species, such as *Arabidopsis* (Xiong et al., 2005; Chen et al., 2008; Biedermann and Hellmann 2010; Lee et al., 2010; Chen and Brandizzi 2012), rice (Raghuvanshi et al., 2001), *Petunia hybrid* (de Vetten et al., 1997), corn (Carey et al., 2004), and pomegranate (Ben-Simhon et al., 2011). The IRA multi copy inhibitor (MSI) belongs to the subfamily of the WD40 repeat protein family. In this study, *MSI* in apple was comprehensively characterized, followed by strict screening and identification, and finally, eight *MdMSI* members were screened (Table 1). There are five *MSI* genes in *Arabidopsis* (Ruggieri et al., 1989) and six in longan (Shen et al., 2019), thus making it clear that there are differences in the number of *MSI* family members among different species. The number of *MSI* family members in apple is more than that in *Arabidopsis*, rice, and longan, indicating a more complex genome in apple.

Through domain analysis and multi-sequence alignment of MSI protein in *Arabidopsis* and apple, it was found that they all have a CAF1C_H4-bd-conserved domain and WD40 domain, indicating that they are highly conservative (Figure 1). CAF1C_H4-bd is a subunit of the CAF-1 complex, and CAF-1 is mainly involved in chromatin formation during DNA replication and damage repair (Winkler et al., 2012). Therefore, it was initially believed that *MSI* genes play an important role in apple growth and stress. A large number of studies have shown that CAF-1 exists conservatively in various species (Yu et al., 2015). Previous studies have shown that MSI1 is also involved in the formation of the CAF-1 complex and MSI1-RBR1 complex (Ach et al., 1997; Exner et al., 2006; Jullien et al., 2008; Kaya et al., 2001). Furthermore, *MdMSIs* were predicted to be located in the nucleus (Table 1). Based on phylogenetic analysis and genetic evolution analysis of *Arabidopsis*, we divided the MdMSI proteins into three classes (Figure 2) (Hennig et al., 2005). The same class of proteins has similar secondary and tertiary structures, indicating that the same class of proteins may have the same function (Table 2 and Figure 4).

Previous studies have shown that *MSI* has been widely reported in reproduction, such as endosperm formation, regulation of flowering process, and pollen development (Bouveret et al., 2006; Pazhouhandeh et al., 2011; Gao et al., 2010; Rodrigues et al., 2010; Dumbliauskas et al., 2011; Mozgová et al., 2010). In addition, the *MSI* family also plays an important role in abiotic stress, especially salt and drought stress (Mehdi et al., 2016; Kenzior and Folk 2015; Kim et al., 2004; Lu et al., 2021). However, *MdMSIs* on abiotic stress have not been described in detail. Tissue expression analysis showed that all *MdMSI* genes were constitutively expressed in the tissues examined, with relatively highly expressed levels in roots, indicating that *MdMSIs* may play an important role in abiotic stress (Figure 7). Similarly, we showed that *MdMSIs* responded to salt, drought stress, and ABA by qRT-PCR (Figure 8). Plants face some abiotic stresses through a large number of transcriptional reactions in stress. However, transcriptional regulation is largely controlled by chromatin (Aditya and Aryadeep, 2017), and the *MSI* gene family is a part of protein complexes involved in the chromatin assembly. Moreover, analysis of the *MdMSI* promoters revealed the presence of a large number of *cis*-acting elements in this family that respond to hormones with adversity, such as ABA. ABA is an important regulator of growth inhibition and plays an irreplaceable role in regulating various stresses (Cutler et al., 2010; Nakashima and Yamaguchi-Shinozaki 2013; Ye et al., 2012). qRT-PCR and promoter analysis further confirmed that apple *MSIs* may play an irreplaceable role in plants facing abiotic stress. In addition, the expression of *MdMSI1-1* was the highest in roots (Figure 7), and *MdMSI1-1* could be significantly induced under salt stress (Figure 8A), indicating that it may be involved in salt stress response because roots are the first plant organs to suffer salt stress (Xing et al., 2021). Here, we obtained transgenic apple calli and ectopical *MdMSI1-1-OE Arabidopsis*. Overexpressing *MdMSI1-1* negatively regulated salt tolerance (Figures 9, 10). It is reported that GmNFYA interacts with GmFVE to jointly regulate salt stress (Lu et al., 2021), and *AtMSI1* has a negative regulatory effect on drought stress (Alexandre et al., 2009).

## Conclusion

In conclusion, eight *MdMSI* genes were identified in the apple. We studied the function of *MdMSI* genes in apple growth and development by bioinformatics, gene expression, and functional analysis. Functional characterization showed that *MdMSIs* may play an important role in salt stress. It will provide a basis for future studies to comprehensively and comparatively analyze the functional characteristics of *MSI* and to deeply investigate aspects of abiotic stress in the apple.

## Data Availability

The original contributions presented in the study are included in the article/Supplementary Material, further inquiries can be directed to the corresponding authors.

## Supplementary Material

Supplementary Table S1 Primers for quantitative real-time PCR.
