# Supplementary material for: Genome-wide Identification, Expression, and Functional Analysis of MdMSI Genes in Apples (Malus domestica Borkh.)
Source: Front Genet. 2022 Mar 3;13:846321. doi: 10.3389/fgene.2022.846321 (PMC8927680; doi:10.3389/fgene.2022.846321)
Supplement: Supplementary file 1 [file Table1.docx]

**Supplementary Materials:**

| **Gene** | **Forward primer (5′-3′)** | **Reverse primer (5′-3′)** |
| --- | --- | --- |
| MdMSI1-1(qRT) | GGAGAGGTTAATCGGGCTCG | AATCAGGACTGCATGCACCA |
| MdMSI1-2(qRT) | AATCAGGACTGCATGCACCA | GGAGAGGTTAATCGGGCTCG |
| MdMSI2(qRT) | GGAGAGGTTAATCGGGCTCG | AATCAGGACTGCATGCACCA |
| MdMSI3-1(qRT) | AATCAGGACTGCATGCACCA | TAGCCGAGTCAGAGACCGAA |
| MdMSI3-2(qRT) | TAGCCGAGTCAGAGACCGAA | GGGGACTCCAGTCTAGAGCA |
| MdMSI4-1(qRT) | GGGGACTCCAGTCTAGAGCA | CTCTCGAATGGCCGTCTCTC |
| MdMSI4-2(qRT) | CTCTCGAATGGCCGTCTCTC | TTTCGGAAATGGCGCCAAAG |
| MdMSI4-3(qRT) | TTTCGGAAATGGCGCCAAAG | CAGGACCAAGAATCGGCTGT |
| MdMSI1-1 | ATGGGCAAAGAC | AGGCTTTGCCGGTTC |
| 18S | ACACGGGGAGGTAGTGACAA | CCTCCAATGGATCCTCGTTA |
| AtACTIN | TTTGGAGCCTGGGACTATGGAT | ACGGGGGAATGGGATGAGAT |

**Table S1:** Primers for quantitative real-time PCR.
